# IL-22 increases proglucagon-expressing enteroendocrine cells via STAT3 in human iPSC-derived intestinal epithelium

**DOI:** 10.1210/endocr/bqag101

**Published:** 2026-09-03

**Authors:** Yu Inoue, Kouya Hattori, Takashige Hamaguchi, Yurina Sueki, Jun Wakabayashi, Yui Funatsu, Hitoshi Watanabe

**Affiliations:** Wellness Science Labs, Meiji Holdings Co., Ltd., Tokyo 192-0919, Japan; Wellness Science Labs, Meiji Holdings Co., Ltd., Tokyo 192-0919, Japan; Wellness Science Labs, Meiji Holdings Co., Ltd., Tokyo 192-0919, Japan; Wellness Science Labs, Meiji Holdings Co., Ltd., Tokyo 192-0919, Japan; Wellness Science Labs, Meiji Holdings Co., Ltd., Tokyo 192-0919, Japan; Wellness Science Labs, Meiji Holdings Co., Ltd., Tokyo 192-0919, Japan; Wellness Science Labs, Meiji Holdings Co., Ltd., Tokyo 192-0919, Japan

**Keywords:** IL-22, GLP-1, enteroendocrine cell, human intestinal organoids, STAT3

## Abstract

Intestinal epithelial differentiation is tightly regulated to maintain tissue homeostasis and systemic metabolic function. Although the intrinsic transcriptional programs governing enteroendocrine cell (EEC) development have been characterized extensively, the role of extrinsic immune signals in directing lineage specification is not completely understood. In particular, how cytokine signaling influences differentiation of EEC subtypes remains unclear. Here, we investigated the role of interleukin-22 (IL-22) in EEC differentiation using human induced pluripotent stem cell-derived intestinal organoids and in vivo models. IL-22 selectively increased proglucagon (*GCG*) expression and the number of glucagon-like peptide-1 (GLP-1)-positive EECs without affecting other EEC markers. This effect was associated with enhanced expression of the lineage-related transcription factors neurogenin 3 and forkhead box A2. Pharmacological inhibition of signal transducer and activator of transcription 3 (STAT3) signaling attenuated the IL-22-induced increases in *GCG* expression and GLP-1-positive cell abundance, indicating a role of STAT3 in mediating these effects. Mechanistically, relatively high expression of IL-22 receptor subunit alpha 1 in undifferentiated epithelial cells was associated with STAT3 activation induced by IL-22. Consistent with these findings, in vivo IL-22 administration increased intestinal *GCG* expression and GLP-1 levels in the circulation and intestinal tissue. Importantly, our findings indicate that IL-22 does not globally promote EEC differentiation but rather selectively directs epithelial lineage commitment toward the GCG-positive EEC fate. These results suggest that IL-22/STAT3 signaling induces human intestinal epithelial differentiation into GCG-positive EECs and may represent a strategy for modulating epithelial cell fate to influence systemic metabolic homeostasis.

The intestinal epithelium is one of the most rapidly renewing tissues in the body, maintained by a balance between stem cell proliferation and lineage-specific differentiation. Continuous epithelial turnover generates multiple specialized cell types, including absorptive enterocytes, goblet cells, Paneth cells, and enteroendocrine cells (EECs), which collectively maintain intestinal homeostasis and systemic metabolic regulation (1, 2). Recent advances have identified key transcription factors, such as atonal homolog 1 (ATOH1), neurogenin 3 (NEUROG3), and forkhead box A2 (FOXA2), as central regulators of intestinal epithelial differentiation (3, 4). However, the extrinsic signals that modulate lineage specification are not completely understood.

Recent studies in intestinal model systems, including organoids, have demonstrated that cytokines can regulate epithelial differentiation programs, including interleukin (IL)-10-mediated restriction of goblet cell expansion, IL-17A-induced secretory lineage commitment, and IL-22-dependent Paneth cell formation, highlighting a strong link between immune signaling and epithelial lineage specification (5-7). EECs represent a rare but functionally critical epithelial lineage that regulates systemic metabolism via hormone secretion. Among these, proglucagon (*GCG*)-expressing L cells produce glucagon-like peptide-1 (GLP-1), a key incretin hormone that enhances insulin secretion and glucose homeostasis (8, 9). The differentiation of GCG-positive cells is controlled by transcription factors including NEUROG3, paired box 6, aristaless-related homeobox, and FOXA2 (3, 10-12). Although intrinsic regulators of enteroendocrine differentiation have been characterized, and microbial metabolites such as short-chain fatty acids and bile acid/GPBAR1 signaling have been shown to influence enteroendocrine and L-cell differentiation (13, 14), the immune-derived epithelial signals that selectively regulate proglucagon-positive enteroendocrine lineage allocation remain incompletely understood.

Among the immune cell-derived cytokines, IL-22 plays a critical role in epithelial barrier maintenance, antimicrobial defense, and tissue repair (15, 16). IL-22 signals primarily via signal transducer and activator of transcription 3 (STAT3) activation in epithelial cells, promoting proliferation and mucosal repair under inflammatory conditions (17, 18). Notably, IL-22 has been reported to enhance GLP-1 production, GLP-1-positive L cell abundance, and STAT3 binding to the *GCG* promoter in mouse models (19). However, whether IL-22 specifically promotes differentiation of human intestinal epithelial cells into the GCG-positive EEC lineage, rather than exerting broader effects on EEC differentiation, remains unclear. Recent single-cell analyses have revealed that EEC differentiation follows heterogeneous and branching lineage trajectories (20), suggesting that extrinsic signals may selectively influence EEC subtype specification. Determining whether IL-22 biases lineage commitment toward the GCG-positive EEC fate is therefore essential for understanding how immune signals shape epithelial endocrine composition and contribute to metabolic regulation.

In this study, we used human induced pluripotent stem cell (iPSC)-derived intestinal epithelial organoids to examine the role of IL-22 in EEC differentiation. IL-22 selectively increased the abundance of GCG-positive cells without affecting other EEC populations, including glucose-dependent insulinotropic polypeptide (*GIP*)-, cholecystokinin (*CCK*)-, and tryptophan hydroxylase 1 (*TPH1*)-expressing cells. Mechanistically, IL-22 activated STAT3 signaling, and STAT3 inhibition abolished these effects. IL-22 also increased the expression of *NEUROG3* and *FOXA2*. Together, these findings indicate that IL-22/STAT3 signaling directs intestinal epithelial differentiation toward the GCG-positive lineage, rather than enhancing EEC differentiation globally, providing insight into immune regulation of epithelial cell fate.

## Materials and methods

### Animals and experimental design

Male C57BL/6J mice were used in all animal experiments. Mice were maintained under specific pathogen-free conditions with ad libitum access to food and water in a temperature- and humidity-controlled environment under a 12-h light/dark cycle. Mice were fed PicoLab R Rodent Diet 20 (LabDiet) for the antibiotic treatment and aging experiments or the CRF-1 diet for the IL-22 administration experiment. Mice were euthanized under isoflurane anesthesia, and the distal one-third of the small intestine was collected as the ileum. Unless otherwise indicated, ileal tissues were stored in RNAlater (Invitrogen) until RNA extraction. All animal experiments were approved by the Meiji Holdings Co., Ltd. Animal Experiment Committee under the following approval numbers: 2019-1000-0035 for the antibiotic treatment experiment, A-2023006 and A-2023007 for the aging experiment, A-2025005 for the IL-22 administration experiment, and A-2026010 for the oral glucose tolerance test (OGTT) and early post-glucose hormone response experiment.

For antibiotic treatment, 9-week-old male mice were assigned to control and antibiotic treatment groups with comparable mean body weights after 2 weeks of acclimation. Ampicillin sodium salt (Nacalai Tesque) was administered in drinking water at 1 g/L for 14 days to the antibiotic treatment group, while the control mice received regular drinking water. Drinking water was replaced twice weekly, and body weight was monitored twice weekly. After a 3-h fast on day 14, ileal tissues were collected. For the aging experiment, ileal tissues were collected from young and old male mice at 12 and 107 weeks of age, respectively, without prior fasting.

For IL-22 administration, 6-week-old male mice were assigned to control and IL-22 treatment groups with comparable mean body weights after 1 week of acclimation. Recombinant mouse IL-22 (PeproTech) was dissolved in PBS and injected intraperitoneally at 0.5 µg per mouse on days 1 and 4; the control mice received PBS. The IL-22 dose was selected based on previous mouse studies using intraperitoneal recombinant IL-22 administration at comparable doses (21). On day 7, portal blood and ileal tissues were collected without prior fasting. Plasma was prepared from EDTA-treated blood and stored at −80 °C until total GLP-1 measurement. In addition to RNAlater-preserved samples for RNA extraction, ileal tissues were snap-frozen in liquid nitrogen for GLP-1 measurement or fixed in 4% paraformaldehyde for immunofluorescence staining.

For the OGTT and early post-glucose hormone response experiments, 6-week-old male mice were assigned to control and IL-22 treatment groups after 1 week of acclimation. Recombinant mouse IL-22 (PeproTech) was dissolved in PBS and injected intraperitoneally at 0.5 µg per mouse on days 1 and 4; control mice received PBS. On day 7, mice were fasted for 5 hours with free access to water. For the OGTT, mice received oral glucose administration (2 g/kg body weight) using a 20% glucose solution (Otsuka Pharmaceutical Factory, Inc.). Blood glucose levels were measured before glucose administration (0 minutes) and at 15, 30, 60, and 120 minutes after glucose administration using a blood glucose meter (LAB Gluco; ForaCare Inc.). For the early post-glucose hormone response experiment, tail-vein blood was collected after the 5-hour fast (0 minutes), followed by oral glucose administration (2 g/kg body weight). Fifteen minutes after glucose administration, mice were euthanized under isoflurane anesthesia and portal blood was collected. Plasma was prepared from EDTA-treated blood and stored at −80 °C until hormone measurements.

### Human iPSC-derived intestinal organoid culture and differentiation

Human intestinal organoids were generated from StemRNA Human iPSC 802-3G cells (Lot. A01RN20; REPROCELL) using the STEMdiff Intestinal Organoid Kit (STEMCELL Technologies) according to the manufacturer's protocol. According to the supplier, the iPSC line was reprogrammed from endothelial progenitor cells derived from ethically sourced blood from a 30-year-old Hispanic female donor. Human iPSCs were maintained on Matrigel hESC-Qualified Matrix-coated plates in mTeSR1 medium (STEMCELL Technologies). Established organoids were embedded in Matrigel Basement Membrane Matrix (CORNING) and maintained in Human IntestiCult Organoid Growth Medium (GM) (STEMCELL Technologies) supplemented with 1× penicillin–streptomycin (Sigma-Aldrich). Organoids were passaged every 7 days using TrypLE Select Enzyme (Gibco) and 10 µM Y-27632 (FUJIFILM Wako Pure Chemical Corporation), a Rho-associated protein kinase (ROCK) inhibitor, to reduce dissociation-induced cell death after passaging. Organoids from passages 6 to 10 were used for experiments. For differentiation, organoids were cultured in GM for 5 days after passaging and then switched to Human IntestiCult Organoid Differentiation Medium (DM) (STEMCELL Technologies) for 4 days.

### Cytokine and inhibitor treatments

For cytokine treatment, organoids were cultured in GM for 5 days after passaging and then maintained in GM or switched to DM with or without recombinant human IL-6, IL-10, IL-17A, or IL-22 (PeproTech) at 10 ng/mL. The cytokine concentration was selected based on previous intestinal organoid studies using recombinant cytokines in the ng/mL range to induce defined epithelial responses (22-24). Organoids were cultured for 4 days unless otherwise indicated, and samples were collected at the time points indicated in the figure legends. For IL-22 timing experiments, organoids were cultured in GM for 5 days after passaging and then switched to DM for 4 days with IL-22 added either throughout the 4-day differentiation period, during the first 2 days only, or during the last 2 days only. For STAT3 inhibition in the quantitative real-time PCR (qPCR) and immunofluorescence analyses, Stattic (Selleck Chemicals), a STAT3 inhibitor, was added at 1 µM simultaneously with IL-22 during differentiation. The concentration of Stattic was selected based on previous studies using 1 µM Stattic to inhibit STAT3 signaling in human intestinal organoids (25). Stattic was dissolved in dimethyl sulfoxide (DMSO), and the same final concentration of DMSO was added to the corresponding control cultures. For short-term STAT3 activation assays, organoids were cultured in DMEM/F-12 for 1 day before cytokine stimulation. Organoids cultured under GM conditions were stimulated with IL-6, IL-10, or IL-22 at 20 ng/mL for 20 minutes to compare cytokine-induced STAT3 activation. To compare IL-22 responsiveness between culture conditions, organoids cultured under GM or DM conditions were stimulated with 20 ng/mL IL-22 for 20 minutes. In these assays, the 0-minute samples were collected immediately before cytokine stimulation. For short-term inhibitor experiments, organoids were pretreated with Stattic or the corresponding DMSO vehicle for 10 minutes before IL-22 stimulation.

### RNA extraction, qPCR, and gene expression quantification

Total RNA was extracted using standard protocols as described previously (26). RNA from mouse ileal tissues was processed using the RNeasy Plus Mini Kit (Qiagen) and that from organoids using the RNeasy Mini Kit (Qiagen). RNA was converted into cDNA using PrimeScript RT Master Mix (TaKaRa). qPCR was performed using TB Green Premix Ex Taq II (TaKaRa) on the 7500 Fast Real-Time PCR System (Applied Biosystems). Relative gene expression was calculated using the 2^−ΔΔCt^ method. *Gapdh* was used as the internal control for the antibiotic treatment and aging experiments, 18S rRNA for organoid experiments, and *Actb* for the IL-22 administration experiment. The primer sequences are listed in Table S1 (27).

Absolute quantification of gene expression was performed using purified qPCR products as standards. qPCR products amplified from organoid cDNA were separated by agarose gel electrophoresis, purified using the QIAquick Gel Extraction Kit (Qiagen), and quantified using the NanoDrop spectrophotometer. Serial dilutions of the purified PCR products were used to generate standard curves. Expression levels were calculated from the standard curves and are presented as arbitrary units.

### Western blotting

Protein lysates were prepared using established methods as described previously (28). Organoids were lysed in CelLytic MT containing protease and phosphatase inhibitors. Protein concentrations were determined using Bradford reagent (Abcam). Equal amounts of protein were separated on NuPAGE 4–12% Bis-Tris gels (Thermo Fisher Scientific) and transferred to polyvinylidene difluoride (PVDF) membranes (Thermo Fisher Scientific). Membranes were blocked with Blocking One or Blocking One-P reagent (Nacalai Tesque) and incubated with antibodies against phospho-STAT3 (Tyr705) (#9145; 1:500; Cell Signaling Technology; RRID:AB_2491009), total STAT3 (#9139; 1:1000; Cell Signaling Technology; RRID:AB_331757), and HRP-conjugated β-actin (#ab20272; 1:20 000; Abcam; RRID:AB_445482). HRP-linked secondary antibodies (#7074 and #7076; 1:1000; Cell Signaling Technology; RRID:AB_2099233 and AB_330924) were used for phospho-STAT3 and total STAT3 detection. Signals were detected using ECL Prime Western Blotting Detection Reagent (Cytiva) and visualized using the LAS-4000 mini imaging system (Cytiva). Phospho-STAT3 signals were normalized to those of total STAT3, and β-actin was used as the loading control.

### Immunofluorescence staining

Immunofluorescence staining of organoids was performed using the BD Cytofix/Cytoperm Fixation/Permeabilization Kit (BD Biosciences) according to the manufacturer's instructions. Organoids were incubated with an anti-GLP-1 antibody (ab22625; 1:200; Abcam; RRID:AB_447206), followed by an Alexa Fluor 555-conjugated secondary antibody (A-31572; 1:500; Invitrogen; RRID:AB_162543). Nuclei were counterstained with Hoechst 33258 (H342; 1:1000; Dojindo). For tissue immunofluorescence staining, ileal tissues were fixed, embedded, and prepared as frozen sections (5 µm). After antigen retrieval using HistoVT One (Nacalai Tesque), sections were incubated with an anti-GLP-1 antibody (ab22625; 1:200; Abcam; RRID:AB_447206), followed by an Alexa Fluor 555-conjugated secondary antibody (A-31572; 1:500; Invitrogen; RRID:AB_162543). Images of both organoids and tissue sections were acquired using the All-in-One Fluorescence Microscope BZ-X800 (Keyence). GLP-1-positive cells in organoids were quantified as the percentage of total nuclei, and the number of images analyzed is indicated in the figure legends. For quantification of tissue sections, GLP-1-positive cells were counted within evaluable villus epithelial regions. Villus epithelial length was measured along the epithelial lining from the crypt–villus junction to the villus tip, excluding crypt regions. Villi without GLP-1-positive cells were included in the denominator when evaluable. GLP-1-positive cell density was expressed as the number of GLP-1-positive cells per millimeter of villus epithelium. Multiple fields were analyzed for each mouse, and the mean value per mouse was used for statistical analysis.

### Measurement of GLP-1 and insulin levels

Total GLP-1 levels in ileal lysates and plasma samples were measured using the Total GLP-1-HS ELISA Kit (YK161; Yanaihara Institute Inc.; RRID:AB_3753759) according to the manufacturer's instructions. Ileal GLP-1 levels were normalized to the total protein concentration. Plasma insulin levels were measured using a Mouse Insulin ELISA Kit (M1104; Morinaga BioScience, Inc.; RRID:AB_2811268) according to the manufacturer's instructions.

### Statistical analysis

Statistical analyses were performed using R software version 4.4.1. Data are presented as the mean ± SEM. Comparisons between two groups were performed using the unpaired or paired two-tailed Student's t-test. Comparisons among multiple groups were performed using one-way ANOVA followed by Tukey's or Dunnett's multiple comparison test, as indicated in the figure legends. Correlations between gene expression levels were assessed using Spearman's rank correlation analysis. For correlation analyses, all samples were pooled regardless of treatment group, with each point representing one biological replicate. *P* < .05 was considered statistically significant. Exact *P* values are shown for comparisons with *P* values between .05 and .10.

## Results

### Antibiotic treatment reduces *Gcg* expression and reveals associations between cytokine expression and *Gcg* expression in vivo

**Figure 1:**
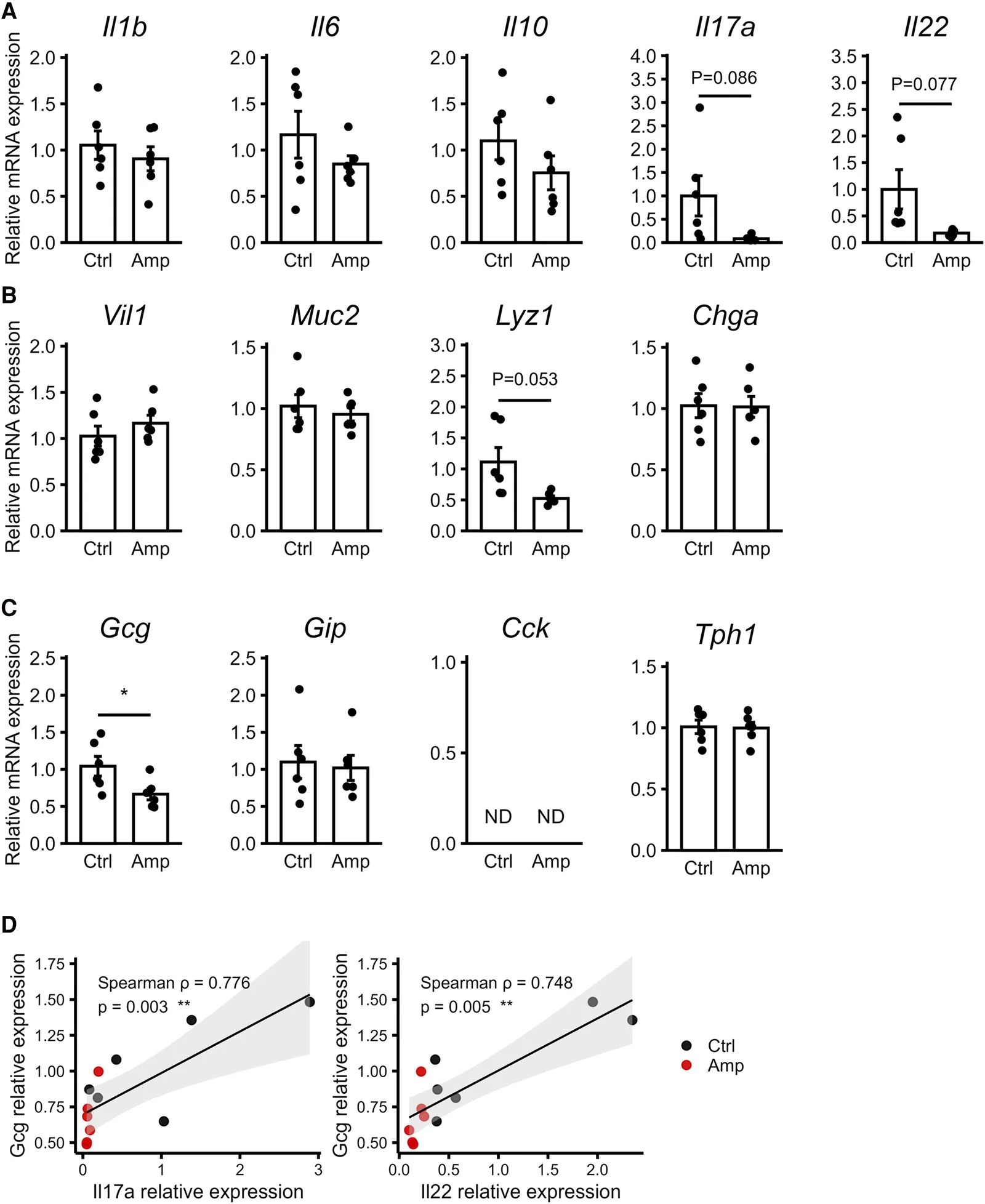
Antibiotic treatment reduces *Gcg* expression and reveals associations between cytokine expression and *Gcg* expression in vivo. (A) Relative mRNA levels of interleukins (*Il1b*, *Il6*, *Il10*, *Il17a*, and *Il22*) in the ileum of control (Ctrl) and ampicillin-treated (Amp) mice. (B) Relative mRNA levels of intestinal epithelial cell markers (*Vil1*, *Muc2*, *Lyz1*, and *Chga*) in the ileum of Ctrl and Amp mice. (C) Relative mRNA levels of enteroendocrine hormone-related genes (*Gcg*, *Gip*, *Cck*, and *Tph1*) in the ileum of Ctrl and Amp mice. (D) Correlations between *Il17a* or *Il22* expression and *Gcg* expression in the ileum. Each dot represents an individual mouse, and Ctrl and Amp mice are indicated by different colors. Correlations were assessed using Spearman's rank correlation analysis using all samples pooled. Linear regression lines are shown for visualization only. Data in (A–C) represent the mean ± SEM, with each dot representing an individual mouse (n = 6 per group). Statistical significance in (A–C) was determined by unpaired two-tailed Student's t-test. *P < .05 vs Ctrl. Exact *P* values are shown for comparisons with *P* values between .05 and .10. ND, not detected.

To explore the potential link between cytokine signaling and EEC differentiation in vivo, we first examined the effects of antibiotics on intestinal cytokine expression and epithelial cell populations. Because the gut microbiota influences interleukin responses (29), antibiotic-induced changes in the intestinal microbiota may alter local interleukin expression and thereby affect epithelial differentiation programs. Mice were treated with the antibiotic ampicillin, and gene expression was analyzed in the ileum. The gene expression of representative interleukins (*Il1b*, *Il6*, *Il10*, *Il17a*, and *Il22*) as well as epithelial cell and EEC markers was assessed. The epithelial cell markers included the absorptive enterocyte marker villin 1 (*Vil1*), the goblet cell marker mucin 2 (*Muc2*), and the Paneth cell marker lysozyme 1 (*Lyz1*). The EEC markers included chromogranin A (*Chga*), as a general enteroendocrine marker, and representative EEC subtype markers, including *Gcg* (L cells), *Gip* (K cells), *Cck* (I cells), and *Tph1* (enterochromaffin cells). Ampicillin treatment did not significantly alter the expression of *Il1β*, *Il6*, or *Il10* (Fig. 1A). Although *Il17a* and *Il22* showed lower mean expression levels after ampicillin treatment, these differences did not reach statistical significance. Among the epithelial cell markers, the expression of *Vil1*, *Muc2*, *Lyz1*, and *Chga* remained unchanged (Fig. 1B). Of the EEC lineage markers, *Gcg* expression was significantly reduced following ampicillin treatment, whereas *Gip* and *Tph1* expression were not affected (Fig. 1C). *Cck* expression was below the detection limit under these conditions in the ileum. Because group-level comparisons after antibiotic treatment cannot determine whether cytokine expression is associated with *Gcg* expression within individual animals, we next performed correlation analyses across individual mice. Correlation analysis using all samples pooled showed positive correlations between *Il17a* or *Il22* expression and *Gcg* expression in the ileum (Fig. 1D). These results suggest that intestinal *Il17a* and *Il22* expression levels are associated with *Gcg* expression in vivo, although this analysis does not establish a causal relationship.

In addition to antibiotic treatment, we examined age-related changes in cytokine expression and epithelial markers by comparing young and aged mice at 12 and 107 weeks of age, respectively. Because aging is associated with alterations in inflammatory cytokines, including IL-1β and IL-6 (30), aged mice were included as an additional exploratory model. Aging was associated with numerically lower mean *Il22* and *Gcg* expression, although these differences did not reach statistical significance, whereas *Il1b* and *Il17a* expression levels were increased (Fig. S1) (27). Altogether, these findings showed that *Il22* exhibited a similar directional pattern to *Gcg* in both the antibiotic and aging datasets, whereas *Il17a* showed model-dependent changes. Together with the correlation analysis in the antibiotic experiment, these exploratory observations provided a rationale for functionally testing candidate cytokines, including IL-17A and IL-22, in the intestinal organoid system.

### IL-22 promotes differentiation toward GCG-positive EECs in human intestinal organoids

**Figure 2:**
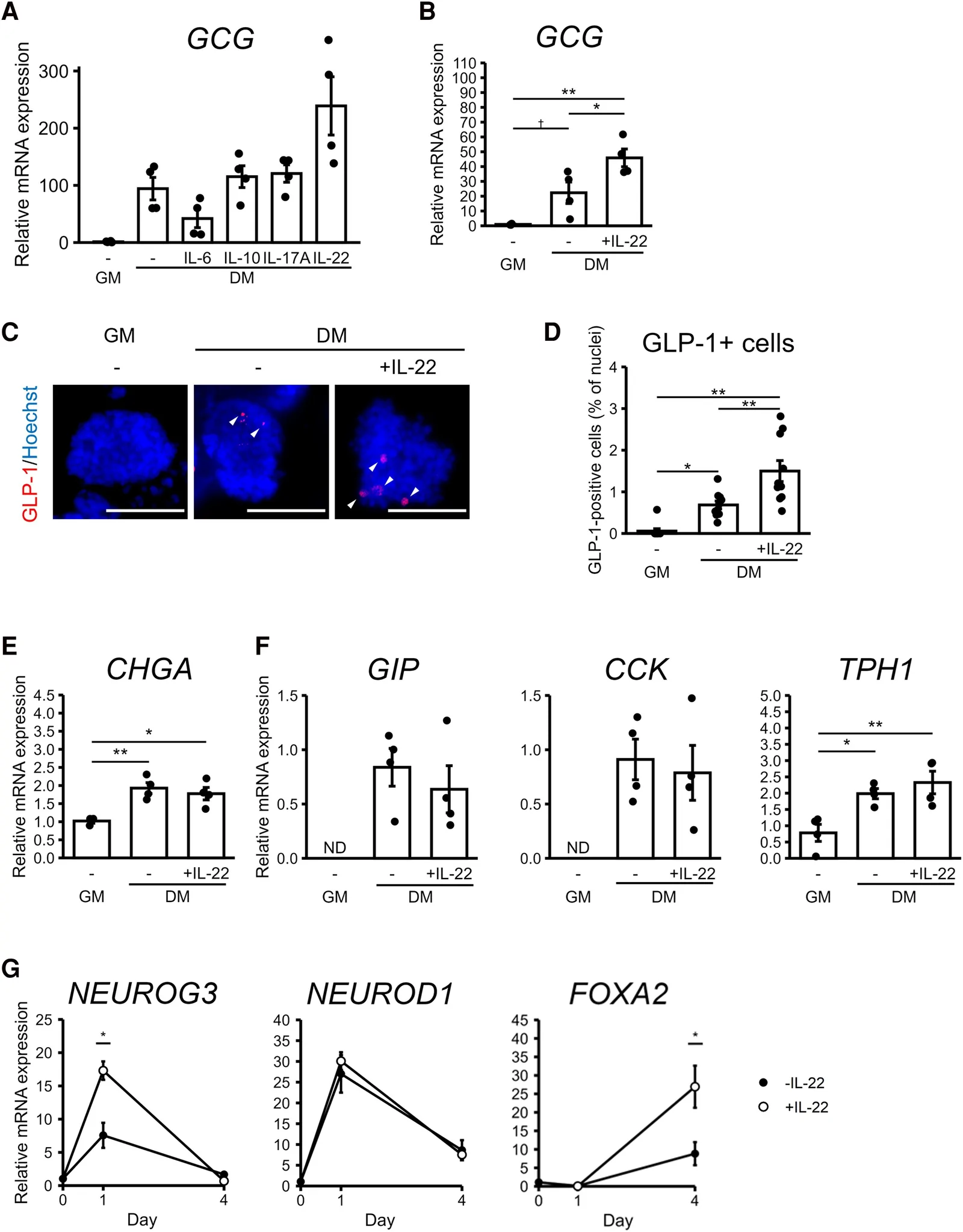
IL-22 promotes differentiation toward GCG-positive EECs in human intestinal organoids. (A) Relative mRNA expression of *GCG* in organoids cultured in GM for 5 days after passage, followed by culture in GM or DM supplemented with interleukins (IL-6, IL-10, IL-17A, or IL-22; 10 ng/mL each) for an additional 4 days. (B) Relative mRNA expression of *GCG* in organoids cultured in GM or DM with or without IL-22 for 4 days. (C) Representative immunofluorescence images of GLP-1-positive cells in organoids cultured in GM or DM with or without IL-22. GLP-1 immunostaining and Hoechst nuclear staining are shown. Arrowheads indicate GLP-1-positive cells. Scale bars, 100 μm. (D) Quantification of GLP-1-positive cells shown as the percentage of total nuclei in organoids cultured as described in (C). (E) Relative mRNA expression of *CHGA* in organoids cultured as described in (B). (F) Relative mRNA expression of enteroendocrine hormone-related genes (*GIP*, *CCK*, and *TPH1*) in organoids cultured as described in (B). (G) Time-course analysis of relative mRNA expression of endocrine differentiation-related transcription factors (*NEUROG3*, *NEUROD1*, and *FOXA2*) during differentiation with or without IL-22. Data represent the mean ± SEM. For (A), (B), (E), and (F), each dot represents an independent biological replicate (n = 4). For (G), each dot represents an independent biological replicate (n = 3). For (D), each dot represents one organoid, and 10 organoids were analyzed per group. For (A), statistical significance was determined by one-way ANOVA followed by Dunnett's multiple comparison test, using the DM without cytokine group as the control. For (B), (D), (E), and (F), statistical significance was determined by one-way ANOVA followed by Tukey's multiple comparison test. For (G), comparisons between the −IL-22 and +IL-22 groups at each time point were performed using unpaired two-tailed Student's t-test. *P < .05, **P < .01. Exact *P* values are shown for comparisons with *P* values between .05 and .10. ND, not detected.

To examine the effects of cytokines on differentiation of iPSC-derived intestinal epithelial organoids toward GCG-positive EECs, we compared the effects of IL-6, IL-10, IL-17A, and IL-22 on *GCG* gene expression in human organoids. In the organoids, *GCG* expression increased after switching from GM to DM, and this increase was most pronounced in the presence of IL-22 (Fig. 2A). Specifically, IL-22 treatment in DM increased *GCG* expression by approximately 2.5-fold compared with DM alone (Fig. 2B). Consistent with the gene expression data, immunostaining using a GLP-1 antibody revealed that the number of GLP-1-positive cells was approximately doubled by IL-22 treatment in DM compared with DM alone (Fig. 2C and 2D). In contrast, IL-22 did not significantly affect the expression of the general enteroendocrine marker *CHGA* or the EEC subtype-specific markers *GIP*, *CCK*, and *TPH1* (Fig. 2E and 2F). To further investigate the effects of IL-22 on EEC differentiation, we analyzed the expression of key transcription factors involved in lineage specification. NEUROG3 is a master regulator of endocrine lineage commitment (10), while NEUROD1 contributes to subsequent differentiation and maturation of EECs (31). FOXA2 is involved in the establishment and maintenance of hormone-producing EECs, including GCG-expressing cells (3). During differentiation, *NEUROG3* and *NEUROD1* expression peaked on day 1 and declined by day 4, whereas *FOXA2* expression was relatively unchanged on day 1 but increased on day 4 (Fig. 2G). IL-22 treatment markedly enhanced *NEUROG3* expression on day 1 and *FOXA2* expression on day 4, suggesting its effects on early endocrine commitment and a possible involvement in later stages of lineage maturation. In contrast, the effect of IL-22 on *NEUROD1* expression was limited. These data suggest that IL-22 promotes differentiation toward GCG-positive EECs without affecting other EEC populations, potentially by enhancing early endocrine commitment and reinforcing GCG lineage-associated transcriptional programs.

### STAT3 signaling is required for the IL-22-associated increase in GCG-positive cell abundance

**Figure 3:**
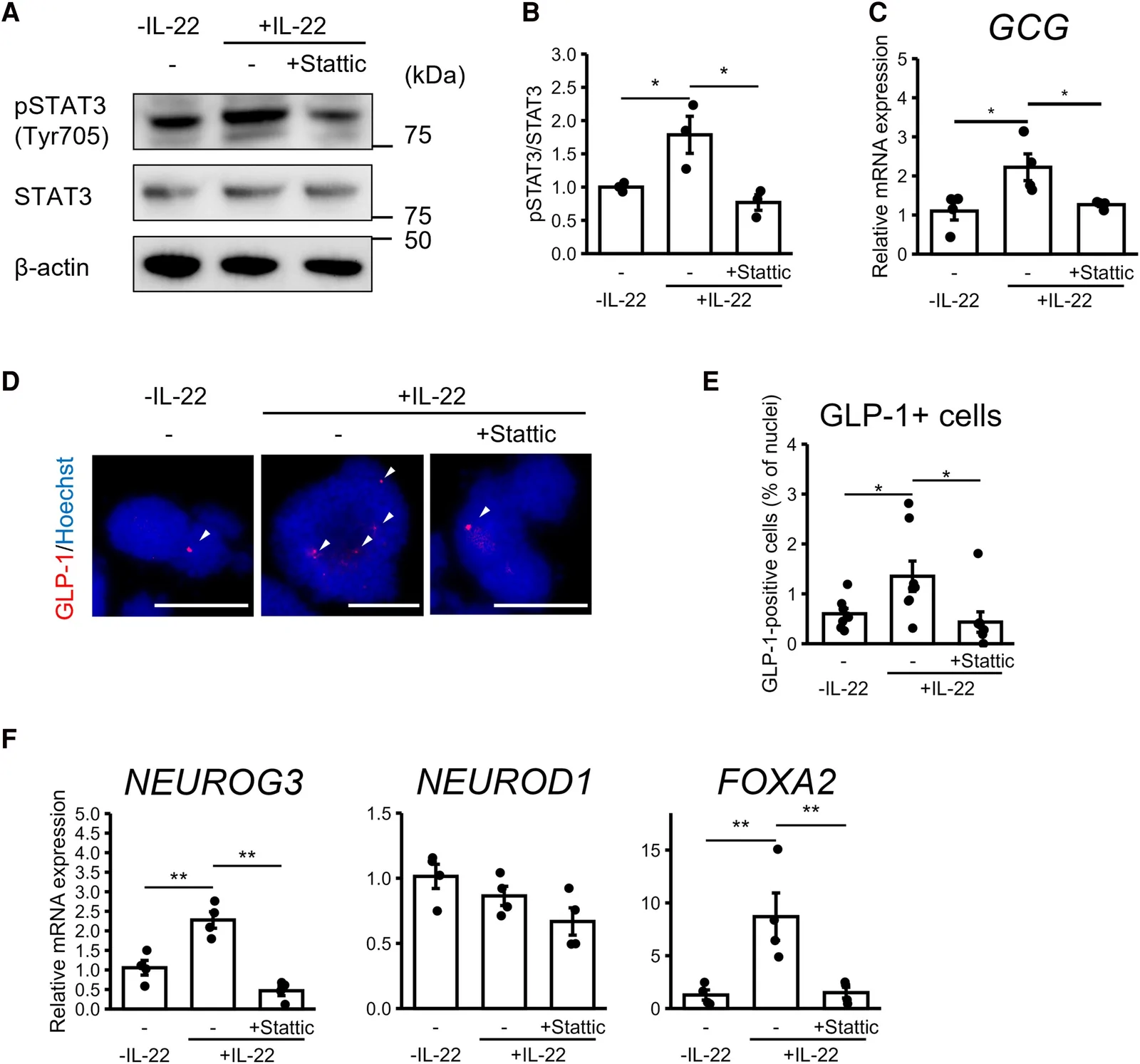
STAT3 signaling is required for IL-22-associated increases in GCG-positive cells. (A) Representative western blot analysis of phosphorylated STAT3 (pSTAT3, Tyr705), total STAT3, and β-actin in intestinal organoids before IL-22 stimulation or after IL-22 stimulation for 20 minutes, with or without 10 minutes pretreatment with Stattic. (B) Quantification of the pSTAT3 (Tyr705) levels, normalized to total STAT3, obtained from (A). (C) Relative mRNA expression of *GCG* in organoids cultured in GM for 5 days after passage and subsequently maintained for 4 days in DM with or without IL-22 (10 ng/mL) and Stattic (1 μM). (D) Representative immunofluorescence images of GLP-1-positive cells and nuclei in organoids cultured under the indicated conditions. Arrowheads indicate GLP-1-positive cells. Scale bars, 100 μm. (E) Quantification of GLP-1-positive cells shown as the percentage of total nuclei in organoids cultured as described in (D). (F) Relative mRNA expression of transcription factors associated with endocrine differentiation in organoids cultured under the conditions described in (C). *NEUROG3* and *NEUROD1* expression was analyzed 1 day after switching to DM, whereas *FOXA2* expression was analyzed after 4 days of culture in DM. Data represent the mean ± SEM. For (B), each dot represents an independent biological replicate (n = 3). For (C) and (F), each dot represents an independent biological replicate (n = 4). For (E), each dot represents one organoid, and 8 organoids were analyzed per group. For (B), (C), and (F), statistical significance was determined by one-way ANOVA with Dunnett's multiple comparison test, using the +IL-22/−Stattic group as the control. *P < .05, **P < .01 vs control.

To determine whether STAT3 signaling mediates the effects of IL-22 on EEC differentiation, we examined STAT3 activation and its functional contribution in human intestinal organoids. IL-22 treatment increased STAT3 phosphorylation in intestinal organoids, whereas co-treatment with the STAT3 inhibitor Stattic markedly attenuated this effect (Fig. 3A). Quantification of the western blot results confirmed that the IL-22-induced increase in phosphorylated STAT3 levels was reduced by Stattic treatment (Fig. 3B). Consistent with this, the IL-22-induced increase in *GCG* expression was significantly reduced by Stattic treatment (Fig. 3C). Immunostaining for GLP-1 showed that IL-22 increased the abundance of GLP-1-positive cells, and quantification confirmed that this increase was attenuated by Stattic (Fig. 3D and 3E). We next examined the expression of key transcription factors involved in EEC differentiation. Stattic significantly attenuated the IL-22-induced increase in *NEUROG3* and *FOXA2* expression, whereas *NEUROD1* expression was not significantly affected (Fig. 3F). These results indicate that STAT3 signaling is required for the IL-22-associated increases in *GCG* expression and GLP-1-positive cell abundance, supporting a role of STAT3 in mediating IL-22-dependent effects on EEC differentiation.

### Differential receptor expression may contribute to selective STAT3 activation by IL-22

**Figure 4:**
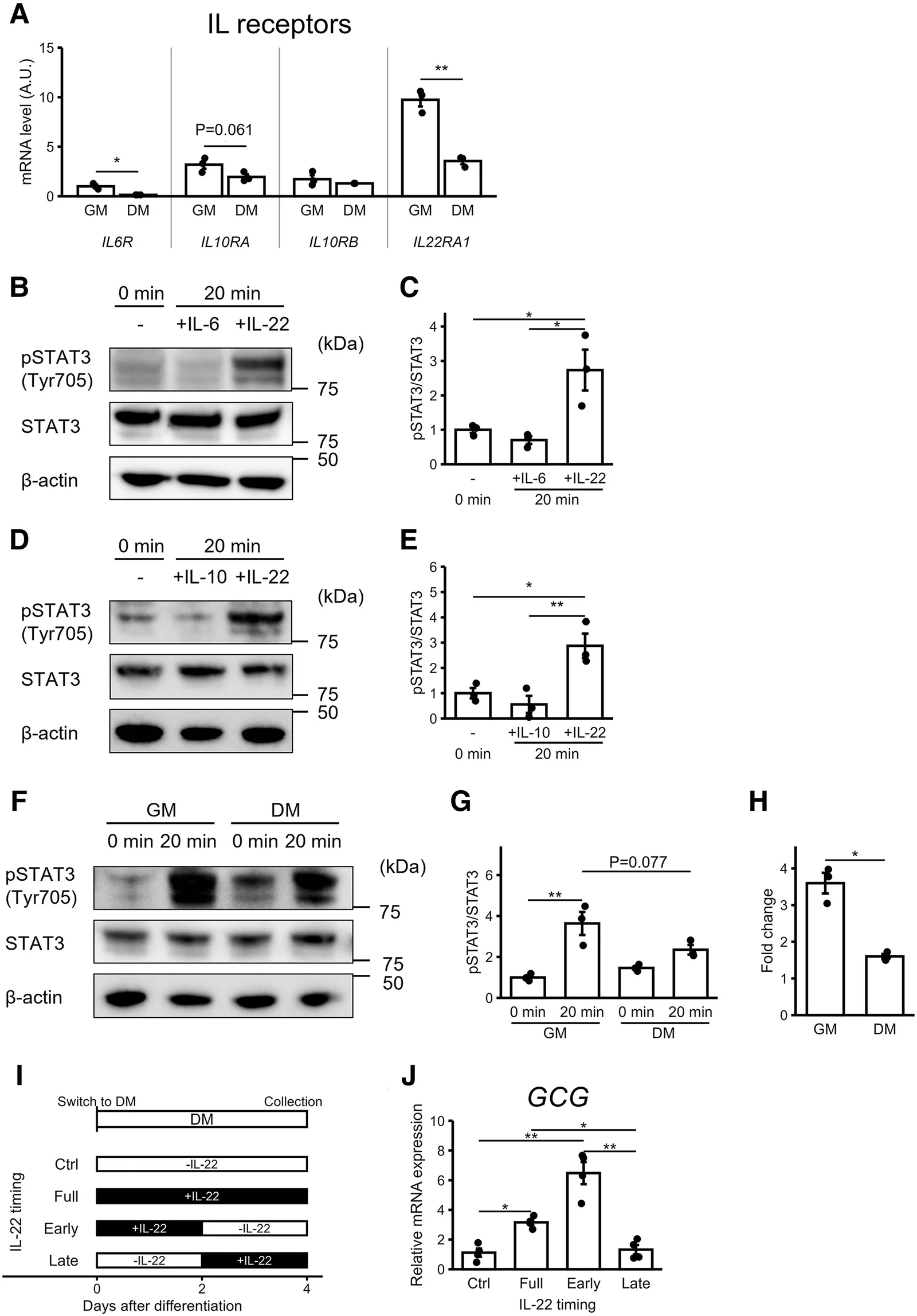
Differential receptor expression may contribute to selective STAT3 activation by IL-22. (A) Absolute mRNA expression levels of interleukin receptor genes (*IL6R*, *IL10RA*, *IL10RB*, and *IL22RA1*) in organoids cultured in GM or DM. Organoids cultured in GM for 5 days after passage were collected as the GM samples, and those further cultured in DM for 4 days were collected as the DM samples. (B) Representative western blot analysis of phosphorylated STAT3 (pSTAT3, Tyr705), total STAT3, and β-actin in organoids cultured under GM conditions before cytokine stimulation (0 minutes) or after stimulation with IL-6 or IL-22 (20 ng/mL) for 20 minutes. (C) Quantification of pSTAT3 (Tyr705) levels normalized to total STAT3 from (B). (D) Representative western blot analysis of pSTAT3 (Tyr705), total STAT3, and β-actin in organoids cultured under GM conditions before cytokine stimulation (0 minutes) or after stimulation with IL-10 or IL-22 (20 ng/mL) for 20 minutes. (E) Quantification of pSTAT3 (Tyr705) levels normalized to total STAT3 from (D). (F) Representative western blot analysis of pSTAT3 (Tyr705), total STAT3, and β-actin in organoids cultured under GM or DM conditions before IL-22 stimulation (0 minutes) or after stimulation with IL-22 (20 ng/mL) for 20 minutes. (G) Quantification of the pSTAT3 (Tyr705) levels, normalized to total STAT3, obtained from (F). (H) Quantification of IL-22-induced STAT3 phosphorylation in organoids cultured under GM or DM conditions. The fold induction was calculated relative to the corresponding 0-minute sample in each condition. (I) Schematic representation of the IL-22 exposure windows during organoid differentiation. Organoids were cultured in GM for 5 days after passage and then switched to DM for 4 days. IL-22 was added either throughout the 4-day DM culture period (Full), during the first 2 days only (Early), or during the last 2 days only (Late). The Ctrl group was cultured in DM without IL-22 for 4 days. (J) Relative mRNA expression of *GCG* in organoids exposed to IL-22 during the indicated time windows of differentiation. Data represent the mean ± SEM. For (A), (C), (E), (G), and (H), each dot represents an independent biological replicate (n = 3). For (J), each dot represents an independent biological replicate (n = 4). For (A), statistical significance was determined by unpaired two-tailed Student's t-test for each gene. For (C), (E), and (G), statistical significance was determined by one-way ANOVA followed by Tukey's multiple comparison test. For (H), statistical significance was determined by paired two-tailed Student's t-test. For (J), statistical significance was determined by one-way ANOVA followed by Tukey's multiple comparison test. *P < .05, **P < .01. Exact *P* values are shown for comparisons with *P* values between .05 and .10.

Based on the involvement of STAT3 signaling in IL-22-associated effects, we next examined the mechanisms underlying selective STAT3 activation by IL-22. Although several cytokines, including IL-6 and IL-10, also signal through STAT3 (32, 33), IL-22 appeared to induce a more pronounced effect on GCG-positive cells in our system. To determine whether differences in cytokine receptor expression contribute to this selectivity, we analyzed the expression of cytokine receptors in organoids cultured under GM and DM conditions. Specifically, we examined the expression of IL-6 receptor (*IL6R*), IL-10 receptor α (*IL10RA*), IL-10 receptor β (*IL10RB*), and IL-22 receptor α1 (*IL22RA1*). In GM, *IL22RA1* showed the highest expression among these receptors, approximately 10-fold higher than *IL6R* expression (Fig. 4A). Compared with GM, culture in DM was associated with lower *IL6R* and *IL22RA1* expression and numerically lower *IL10RA* expression, whereas *IL10RB* expression was largely unchanged. To assess whether these differences in receptor expression are associated with STAT3 activation, we compared STAT3 phosphorylation in response to IL-6 and IL-22 stimulation in intestinal organoids. IL-22 stimulation markedly increased STAT3 phosphorylation, whereas IL-6 stimulation induced minimal STAT3 phosphorylation (Fig. 4B and 4C). Similarly, IL-10 stimulation did not markedly induce STAT3 phosphorylation under the same conditions, in contrast to the robust response induced by IL-22 (Fig. 4D and 4E). These results suggest that IL-22 selectively induces STAT3 activation more efficiently than other STAT3-related cytokines in this organoid system.

Furthermore, we compared IL-22-induced STAT3 phosphorylation in organoids cultured in GM vs DM. IL-22 treatment significantly increased STAT3 phosphorylation in GM, whereas this response was weaker in DM (Fig. 4F and 4G). Although the absolute level of STAT3 phosphorylation after IL-22 stimulation was higher in mean value in GM than in DM, fold-change analysis demonstrated that IL-22-induced STAT3 activation was significantly greater in GM (Fig. 4H). These results suggest that undifferentiated organoids cultured under GM conditions exhibit higher responsiveness to IL-22.

Finally, to examine whether the timing of IL-22 exposure influences *GCG* induction, we compared continuous IL-22 treatment throughout the 4-day differentiation period with IL-22 exposure limited to either the first or last 2 days of differentiation (Fig. 4I). IL-22 treatment during the first 2 days of differentiation (Early) induced the highest *GCG* expression, exceeding that observed with continuous 4-day IL-22 treatment (Full). In contrast, IL-22 treatment during the last 2 days of differentiation (Late) failed to induce *GCG* expression to the same extent and resulted in significantly lower expression than both the Early and Full treatment groups (Fig. 4J). These results indicate that *GCG* induction by IL-22 is most prominent when IL-22 is present during the early phase of differentiation, consistent with the higher *IL22RA1* expression observed before DM-induced differentiation (Fig. 4A).

### IL-22 administration increases *Gcg* expression and circulating GLP-1 levels in vivo

**Figure 5:**
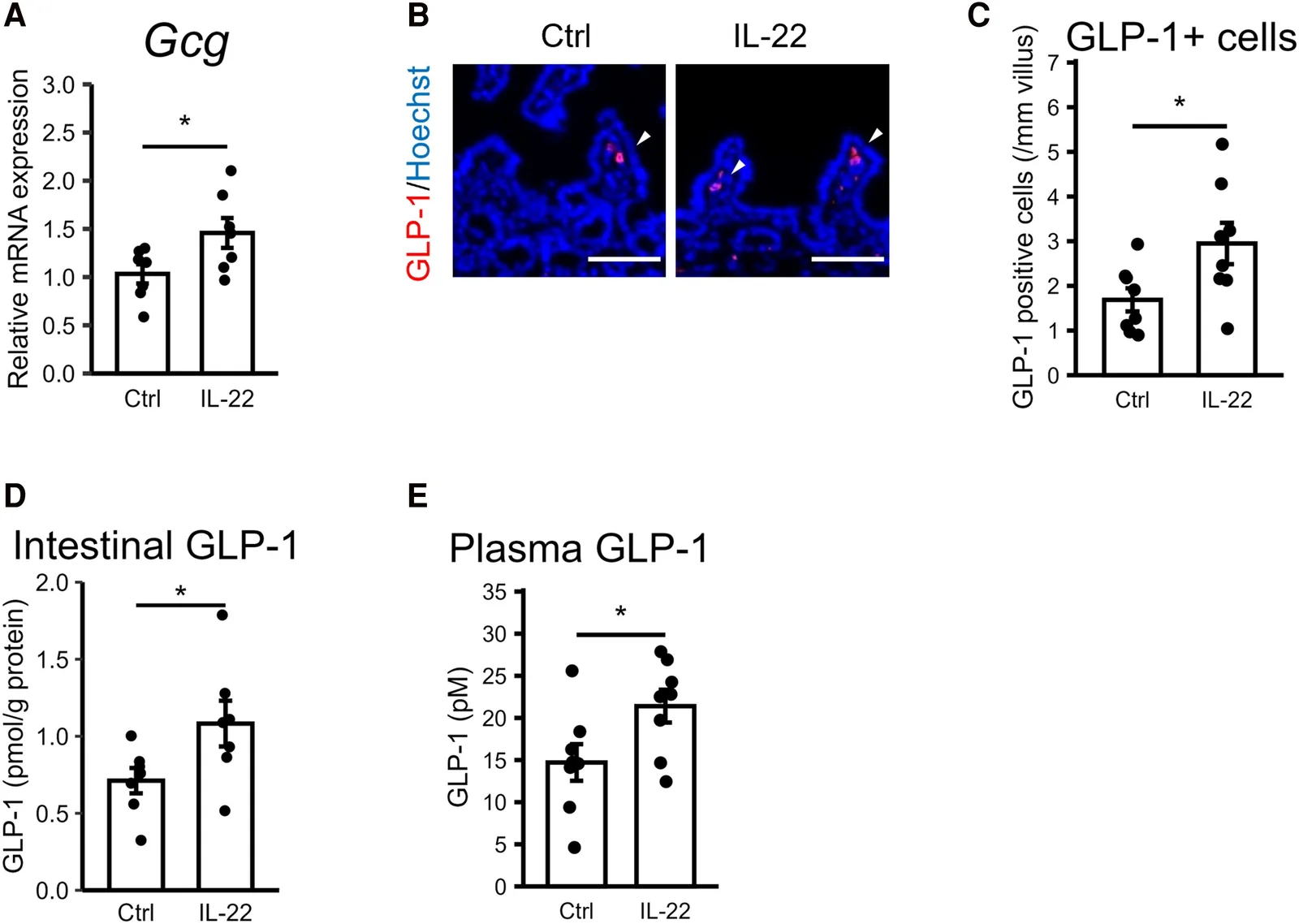
IL-22 administration increases *Gcg* expression and circulating GLP-1 levels in vivo. C57BL/6 male mice (6 weeks old) received intraperitoneal IL-22 (0.5 μg) on days 1 and 4 and were analyzed on day 7. (A) Relative mRNA expression of *Gcg* in the ileum. (B) Representative immunofluorescence images of GLP-1-positive cells and nuclei in the ileum. Arrowheads indicate GLP-1-positive cells. Scale bars, 100 μm. (C) Quantification of GLP-1-positive cells in ileal sections, expressed as the number of GLP-1-positive cells per millimeter of villus epithelium. Villus epithelial length was measured within evaluable villus regions in each field, excluding crypt regions. (D) Total GLP-1 levels in ileal tissue measured by ELISA. (E) Total GLP-1 levels in plasma measured by ELISA. For (A) and (D), n = 7 mice per group; for (C) and (E), n = 8 mice per group. Each dot represents an individual mouse. Data are represented as the mean ± SEM. Statistical significance was determined by unpaired two-tailed Student's t-test. *P < .05.

Having established that IL-22 promotes GCG-positive EEC differentiation through STAT3 signaling in organoids, we next investigated whether these effects occur in vivo to assess their physiological relevance. To examine the effect of IL-22 on GCG expression in vivo, C57BL/6 mice were intraperitoneally administered IL-22 (0.5 μg) on days 1 and 4, and tissues were analyzed on day 7. IL-22 administration increased *Gcg* expression in the intestine (Fig. 5A). Consistent with this, immunostaining revealed an increased number of GLP-1-positive cells in intestinal tissue following IL-22 treatment (Fig. 5B). Quantification of GLP-1-positive cells normalized to villus epithelial length confirmed an increase in GLP-1-positive cell density after IL-22 administration (Fig. 5C). Furthermore, the GLP-1 level was significantly elevated in the intestine (Fig. 5D) and portal plasma (Fig. 5E) following IL-22 administration, as measured by ELISA. Together, these results indicate that IL-22 enhances *Gcg* expression and GLP-1 production in vivo, supporting its role in promoting GCG-positive EEC function under physiological conditions.

### IL-22 is associated with increased basal and early post-glucose GLP-1/insulin levels in vivo

**Figure 6:**
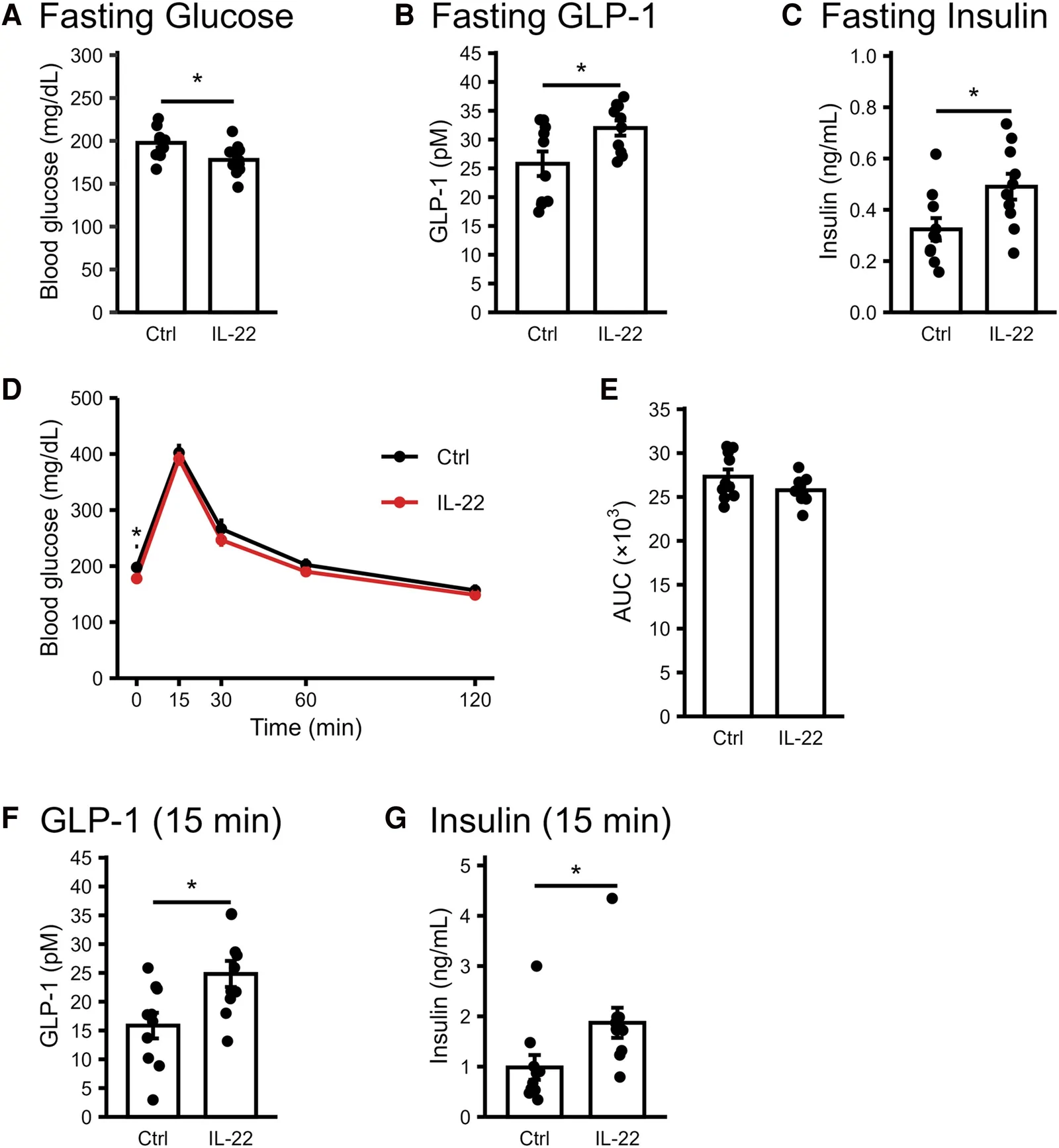
IL-22 is associated with increased basal and early post-glucose GLP-1/insulin levels in vivo. C57BL/6 male mice (6 weeks old) received intraperitoneal injections of IL-22 (0.5 μg per mouse) or PBS (Ctrl) on days 1 and 4 and were analyzed on day 7 after a 5-hour fast. (A) Blood glucose levels after a 5-hour fast. (B) Total GLP-1 levels in fasting plasma collected from the tail vein. (C) Insulin levels in fasting plasma collected from the tail vein. (D) OGTT. Blood glucose levels were measured before glucose administration (0 minutes) and at 15, 30, 60, and 120 minutes after oral glucose administration (2 g/kg body weight). (E) AUC calculated from the blood glucose profiles shown in (D). (F) Total GLP-1 levels in portal plasma collected 15 minutes after oral glucose administration. (G) Insulin levels in portal plasma collected 15 minutes after oral glucose administration. Each dot represents an individual mouse (n = 10 mice per group). Data are represented as the mean ± SEM. Statistical significance was determined by unpaired two-tailed Student's t-test. For (D), comparisons between groups were performed at each time point. *P < .05.

Having shown that IL-22 increases intestinal *Gcg* expression and GLP-1 levels in vivo, we next examined whether this effect was associated with altered glucose handling and endocrine responses during an OGTT. Chow-fed mice were fasted for 5 hours before oral glucose administration. At baseline after the 5-hour fast (0 minutes), IL-22-treated mice showed significantly lower blood glucose levels than PBS-treated control mice (Fig. 6A). In contrast, fasting plasma total GLP-1 and insulin levels, measured in tail-vein plasma, were significantly higher in IL-22-treated mice than in control mice (Fig. 6B and 6C). During the OGTT, blood glucose levels after oral glucose administration were not significantly different between control and IL-22-treated mice, and the area under the curve for blood glucose was not significantly altered by IL-22 treatment (Fig. 6D and 6E). We next examined early post-glucose hormone levels by collecting portal plasma 15 minutes after oral glucose administration. Portal plasma total GLP-1 and insulin levels at this early post-glucose time point were significantly higher in IL-22-treated mice than in control mice (Fig. 6F and 6G). Although IL-22 treatment did not significantly improve overall glucose tolerance under chow-fed conditions, these findings indicate that IL-22 is associated with lower fasting blood glucose and increased basal and early post-glucose GLP-1/insulin endocrine responses in vivo.

## Discussion

In this study, we investigated the role of IL-22 in EEC differentiation using human iPSC-derived intestinal epithelial organoids and complementary in vivo analyses. IL-22 increased *GCG* expression and the number of GLP-1-positive cells without significantly affecting other EEC populations, including *GIP*, *CCK*, and *TPH1*. These effects were accompanied by enhanced expression of the lineage-associated transcription factors *NEUROG3* and *FOXA2* and were attenuated by pharmacological inhibition of STAT3, indicating that STAT3 signaling mediates IL-22-associated responses. Differential expression of cytokine receptors, including relatively high *IL22RA1* expression in undifferentiated epithelial cells, may contribute to the selective activation of this pathway. Consistent with these findings, IL-22 administration in vivo increased intestinal *Gcg* expression and GLP-1 levels in intestinal tissue and portal plasma. Together, these observations suggest that IL-22/STAT3 signaling is associated with increased GCG-positive EEC abundance and GLP-1 production and may influence the distribution of EEC lineages in the intestinal epithelium.

Our findings should also be considered in the context of previous studies showing that luminal and microbiota-related signals can regulate EEC and L-cell differentiation. Petersen et al demonstrated that short-chain fatty acids promote EEC differentiation and increase GLP-1 release in mouse and human intestinal organoids (13). Similarly, Lund et al showed that bile acids induce L-cell differentiation through GPBAR1 signaling and enhance GLP-1-associated glucose responses (14). These studies established that microbial metabolites and nutrient-related GPCR signaling can influence the differentiation and function of GLP-1-producing L cells.

A recent study demonstrated that IL-22 enhances GLP-1 production, GLP-1-positive L cell abundance, and glucose homeostasis via STAT3-dependent regulation of the *GCG* promoter (19). In agreement with this report, we observed that IL-22 increased intestinal *GCG* expression and GLP-1 levels in vivo. However, the present study extends these findings by demonstrating that IL-22 directly influences epithelial lineage allocation in a human iPSC-derived intestinal organoid system, providing evidence that cytokine signaling can bias EEC subtype specification at the level of epithelial differentiation. In particular, IL-22 increased *GCG* expression and GLP-1-positive cell abundance without significantly affecting *CHGA*, *GIP*, *CCK*, or *TPH1*, suggesting that IL-22 influences the distribution of EEC populations rather than simply increasing EEC differentiation globally. Building on these findings, our study identifies IL-22 as an immune-derived epithelial signal that promotes GCG-positive EEC differentiation in human iPSC-derived intestinal organoids. Thus, rather than suggesting that IL-22 is the only external regulator of L-cell differentiation, our data extend this concept by linking cytokine-dependent epithelial STAT3 signaling to proglucagon-positive lineage allocation. The use of human iPSC-derived intestinal epithelial organoids is an important aspect of this study. Organoid systems allow evaluation of epithelial-intrinsic responses under defined conditions, avoiding indirect effects mediated by immune cells, the microbiota, or systemic metabolism. Human intestinal organoid technologies have been widely used to model epithelial differentiation and disease (34-36). More recent studies have generated human EECs from stem cell-derived systems (37, 38). In this context, these results suggest that IL-22 can act directly on human intestinal epithelial cells to increase the abundance of GCG-positive EECs, providing a complementary perspective to in vivo studies.

A central implication of our data is that cytokines function as regulators of epithelial lineage allocation. Previous studies have demonstrated that immune cell-derived signals influence intestinal epithelial differentiation programs. IL-17RA signaling in intestinal stem cells promotes ATOH1 expression and secretory lineage commitment (6), while IL-22 has been implicated in epithelial regeneration and Paneth cell formation (7, 16). These findings indicate that cytokine signaling interfaces with epithelial differentiation programs. Our study extends this framework to EEC differentiation by showing that IL-22 is associated with increased GCG-positive cell abundance in a human epithelial model. Mechanistically, our findings support a role of STAT3 signaling in IL-22-associated effects. IL-22 robustly induced STAT3 phosphorylation, and pharmacological inhibition of STAT3 attenuated IL-22-induced increases in *GCG* expression and GLP-1-positive cell abundance. These results are consistent with the established function of IL-22 in activating STAT3 to regulate epithelial repair and host defense (15, 17, 18). They are also consistent with recent evidence that STAT3 directly regulates *GCG* transcription (19). Together, these findings suggest that STAT3 links cytokine signaling to both hormone gene regulation and differentiation-associated transcriptional programs.

Our receptor expression data provide a potential explanation for the selective effect of IL-22. Although multiple cytokines can activate STAT3, the magnitude and context of activation depend on receptor expression and cellular state. This is particularly relevant for the comparison between IL-6 and IL-22. Classical IL-6 signaling requires membrane-bound IL-6R, and previous work has shown that whereas IL-6 induces STAT3 phosphorylation in colonic cancer cell lines, primary nonmalignant intestinal organoids respond poorly to classical IL-6 signaling (39). In contrast, IL-22 responsiveness is largely determined by epithelial expression of IL-22RA1, which is abundantly expressed across intestinal epithelial lineages (40). Consistent with these observations, we found that *IL22RA1* expression was relatively high in undifferentiated epithelial cells and that IL-22 induced stronger STAT3 phosphorylation than did IL-6. Moreover, IL-22-induced STAT3 phosphorylation was more pronounced in GM than in DM, particularly when assessed as fold induction, suggesting that undifferentiated epithelial cells exhibit enhanced responsiveness to IL-22 signaling. This observation indicates that the responsiveness to IL-22 may be highest during early differentiation stages, when lineage decisions are being established. This interpretation is further supported by our time-window experiment, in which IL-22 exposure during the first 2 days of differentiation induced stronger *GCG* expression than IL-22 exposure during the last 2 days. These findings help reconcile the apparent discrepancy between the reduced *IL22RA1* expression and attenuated STAT3 phosphorylation observed under DM conditions and the ability of IL-22 to promote *GCG* induction during the differentiation protocol. Rather than acting primarily on late-stage differentiated cells, IL-22 appears to exert its strongest effect during an early differentiation window when *IL22RA1* expression and STAT3 responsiveness are relatively preserved. Notably, early IL-22 exposure induced *GCG* expression at least as strongly as continuous IL-22 exposure throughout the differentiation period. This suggests that sustained IL-22 stimulation is not required for *GCG* induction once early differentiation-associated signaling has been initiated. The slightly lower response observed with continuous IL-22 treatment may reflect stage-dependent changes in epithelial responsiveness during prolonged differentiation, although the underlying mechanism remains to be determined. This is consistent with the broader concept that epithelial differentiation is tightly coupled to developmental stage-specific signaling inputs (1, 2). The observed changes in *NEUROG3* and *FOXA2* expression further support a differentiation-related mechanism. NEUROG3 is a master regulator of EEC lineage specification (10, 11, 41, 42), and FOXA factors are critical for intestinal epithelial differentiation and EEC identity (3). In our system, IL-22 enhanced *NEUROG3* expression at early stages and *FOXA2* expression at later stages, suggesting effects on both lineage commitment and maturation. The limited effect on *NEUROD1* suggests that IL-22 does not uniformly accelerate all stages of EEC differentiation (31) but rather influences specific transcriptional nodes associated with GCG-positive cell development. The observation that *CHGA* expression was not increased by IL-22 is also informative. CHGA is a general enteroendocrine marker, whereas GCG, GIP, CCK, and TPH1 are markers of distinct EEC subtypes. The selective increase in *GCG* without broad induction of the other markers indicates that IL-22 may alter the relative distribution of EEC subtypes rather than expanding the EEC compartment as a whole. This interpretation is consistent with recent single-cell analyses showing that EEC differentiation involves heterogeneous and dynamic lineage trajectories (20, 43-45).

The IL-22-associated increase in GCG-positive EEC differentiation observed in organoid models prompted us to examine its relevance in vivo. IL-22 administration increased intestinal *Gcg* expression and GLP-1 levels in intestinal tissue and portal plasma. Given that GLP-1 is secreted into the portal circulation before systemic degradation, increased portal GLP-1 likely reflects enhanced intestinal secretion. These findings align with the established role of GLP-1 as an incretin hormone that regulates glucose homeostasis (8, 9, 46). In support of this interpretation, IL-22-treated mice showed lower fasting blood glucose levels and higher fasting total GLP-1 and insulin levels. Moreover, portal plasma total GLP-1 and insulin levels at 15 minutes after oral glucose administration were increased in IL-22-treated mice. Although these endocrine changes were not accompanied by a significant improvement in overall glucose tolerance under chow-fed conditions, they suggest that IL-22-induced GLP-1 elevation is associated with altered basal and early post-glucose endocrine responses in vivo. More broadly, recent studies have demonstrated that altering intestinal epithelial differentiation toward EEC lineages can improve glucose metabolism, highlighting the physiological relevance of lineage composition in metabolic regulation (47, 48). Taken together, these findings suggest that modulation of intestinal epithelial cell fate may influence systemic metabolic homeostasis. Regulation of GCG-positive EEC differentiation and GLP-1 production may be one example of how epithelial lineage composition influences systemic metabolic homeostasis.

Our findings may have physiological relevance in the context of a gut microbiota–IL-22–GLP-1 axis. The intestinal microbiota and microbial metabolites are known to regulate mucosal IL-22 production (29, 49). The gut microbiota has also been implicated in the regulation of GLP-1 production and metabolic homeostasis. Recent evidence suggests that butyrate can enhance IL-22 production and increase GLP-1 production in an IL-22R-dependent manner (19). Thus, our data provide a potential epithelial mechanism by which microbiota-dependent IL-22 signaling may regulate GCG-positive EEC abundance and intestinal GLP-1 production. However, because microbiota composition and microbial metabolites were not directly assessed in this study, this axis should be examined in future studies (29).

Finally, several limitations should be considered. Lineage tracing was not performed in this study, and thus we cannot formally distinguish increased differentiation from expansion of preexisting GLP-1-positive cells. However, the selective increase in *GCG* expression without concomitant changes in other EEC markers supports the interpretation that IL-22 preferentially biases differentiation toward the GCG-positive lineage. GLP-1 secretion from organoids was not measured directly. Nevertheless, the observed increases in intestinal and portal plasma GLP-1 levels in vivo support the physiological relevance of IL-22-associated effects on EEC function. STAT3 involvement was assessed using pharmacological inhibition, and genetic approaches will be required to further strengthen the mechanistic conclusions. In addition, the regulation of *IL22RA1* expression during differentiation remains to be elucidated.

In summary, our findings suggest that IL-22/STAT3 signaling promotes GCG-positive EEC abundance and GLP-1 production, providing new insight into how immune cell-derived signals regulate human intestinal epithelial differentiation. More broadly, these results highlight cytokine-driven lineage allocation as a previously underappreciated mechanism linking immune signaling to systemic metabolic homeostasis.

## Data Availability

Supplementary materials are available in Zenodo (27). Some or all other datasets generated during and/or analyzed during the current study are not publicly available but are available from the corresponding author on reasonable request.

## References

[1] Barker N . Adult intestinal stem cells: critical drivers of epithelial homeostasis and regeneration. Nat Rev Mol Cell Biol. 2014;15(1):19‐33. 24326621 10.1038/nrm3721

[2] Clevers H . The intestinal crypt, a prototype stem cell compartment. Cell. 2013;154(2):274‐284. 23870119 10.1016/j.cell.2013.07.004

[3] Ye DZ, Kaestner KH. Foxa1 and Foxa2 control the differentiation of goblet and enteroendocrine L- and D-cells in mice. Gastroenterology. 2009;137(6):2052‐2062. 19737569 10.1053/j.gastro.2009.08.059 PMC2789913

[4] Noah TK, Donahue B, Shroyer NF. Intestinal development and differentiation. Exp Cell Res. 2011;317(19):2702‐2710. 21978911 10.1016/j.yexcr.2011.09.006 PMC3210330

[5] Morales RA, Rabahi S, Diaz OE, et al Interleukin-10 regulates goblet cell numbers through Notch signaling in the developing zebrafish intestine. Mucosal Immunol. 2022;15(5):940‐951. 35840681 10.1038/s41385-022-00546-3 PMC9385495

[6] Lin X, Gaudino SJ, Jang KK, et al IL-17RA-signaling in Lgr5(+) intestinal stem cells induces expression of transcription factor ATOH1 to promote secretory cell lineage commitment. Immunity. 2022;55(2):237‐253.e8. 35081371 10.1016/j.immuni.2021.12.016 PMC8895883

[7] He GW, Lin L, DeMartino J, et al Optimized human intestinal organoid model reveals interleukin-22-dependency of paneth cell formation. Cell Stem Cell. 2022;29(9):1333‐1345.e6. 36002022 10.1016/j.stem.2022.08.002 PMC9438971

[8] Drucker DJ . Mechanisms of action and therapeutic application of glucagon-like peptide-1. Cell Metab. 2018;27(4):740‐756. 29617641 10.1016/j.cmet.2018.03.001

[9] Holst JJ . The physiology of glucagon-like peptide 1. Physiol Rev. 2007;87(4):1409‐1439. 17928588 10.1152/physrev.00034.2006

[10] Jenny M, Uhl C, Roche C, et al Neurogenin3 is differentially required for endocrine cell fate specification in the intestinal and gastric epithelium. EMBO J. 2002;21(23):6338‐6347. 12456641 10.1093/emboj/cdf649 PMC136953

[11] Schonhoff SE, Giel-Moloney M, Leiter AB. Neurogenin 3-expressing progenitor cells in the gastrointestinal tract differentiate into both endocrine and non-endocrine cell types. Dev Biol. 2004;270(2):443‐454. 15183725 10.1016/j.ydbio.2004.03.013

[12] Beucher A, Gjernes E, Collin C, et al The homeodomain-containing transcription factors Arx and Pax4 control enteroendocrine subtype specification in mice. PLoS One. 2012;7(5):e36449. 22570716 10.1371/journal.pone.0036449 PMC3343025

[13] Petersen N, Reimann F, Bartfeld S, et al Generation of L cells in mouse and human small intestine organoids. Diabetes. 2014;63(2):410‐420. 24130334 10.2337/db13-0991 PMC4306716

[14] Lund ML, Sorrentino G, Egerod KL, et al L-cell differentiation is induced by bile acids through GPBAR1 and paracrine GLP-1 and serotonin signaling. Diabetes. 2020;69(4):614‐623. 32041793 10.2337/db19-0764 PMC7224989

[15] Sabat R, Ouyang W, Wolk K. Therapeutic opportunities of the IL-22-IL-22R1 system. Nat Rev Drug Discov. 2014;13(1):21‐38. 24378801 10.1038/nrd4176

[16] Lindemans CA, Calafiore M, Mertelsmann AM, et al Interleukin-22 promotes intestinal-stem-cell-mediated epithelial regeneration. Nature. 2015;528(7583):560‐564. 26649819 10.1038/nature16460 PMC4720437

[17] Pickert G, Neufert C, Leppkes M, et al STAT3 links IL-22 signaling in intestinal epithelial cells to mucosal wound healing. J Exp Med. 2009;206(7):1465‐1472. 19564350 10.1084/jem.20082683 PMC2715097

[18] Zheng Y, Valdez PA, Danilenko DM, et al Interleukin-22 mediates early host defense against attaching and effacing bacterial pathogens. Nat Med. 2008;14(3):282‐289. 18264109 10.1038/nm1720

[19] Kim CW, Ahn JH, Lee BR, et al Intestinal interleukin-22 enhances GLP-1 production via the STAT3 pathway to improve glucose homeostasis during high-fat diet induced obesity in a study with male mice. Nat Commun. 2026;17(1):3009. 41723134 10.1038/s41467-026-69734-0 PMC13035814

[20] Gehart H, van Es JH, Hamer K, et al Identification of enteroendocrine regulators by real-time single-cell differentiation mapping. Cell. 2019;176(5):1158‐1173.e16. 30712869 10.1016/j.cell.2018.12.029

[21] Nong H, Yuan H, Lin Y, et al IL-22 promotes occludin expression by activating autophagy and treats ulcerative colitis. Mol Cell Biochem. 2024;479(6):1443‐1450. 37440121 10.1007/s11010-023-04806-z

[22] Wakai M, Hayashi R, Ueno Y, et al Promoting mechanism of serum amyloid a family expression in mouse intestinal epithelial cells. PLoS One. 2022;17(3):e0264836. 35303008 10.1371/journal.pone.0264836 PMC8932556

[23] Chen Y, Vandereyken M, Newton IP, Moraga I, Näthke IS, Swamy M. Loss of adenomatous polyposis coli function renders intestinal epithelial cells resistant to the cytokine IL-22. PLoS Biol. 2019;17(11):e3000540. 31770366 10.1371/journal.pbio.3000540 PMC6903767

[24] Pavlidis P, Tsakmaki A, Pantazi E, et al Interleukin-22 regulates neutrophil recruitment in ulcerative colitis and is associated with resistance to ustekinumab therapy. Nat Commun. 2022;13(1):5820. 36192482 10.1038/s41467-022-33331-8 PMC9530232

[25] Jung KB, Kwon O, Lee MO, et al Blockade of STAT3 causes severe in vitro and in vivo maturation defects in intestinal organoids derived from human embryonic stem cells. J Clin Med. 2019;8(7):976. 31277507 10.3390/jcm8070976 PMC6678857

[26] Watanabe H, Asahara SI, Son J, McKimpson WM, de Cabo R, Accili D. Cyb5r3 activation rescues secondary failure to sulfonylurea but not β-cell dedifferentiation. PLoS One. 2024;19(2):e0297555. 38335173 10.1371/journal.pone.0297555 PMC10857566

[27] Inoue Y, Hattori K, Hamaguchi T, et al 2026. Supplementary material for IL-22 increases proglucagon-expressing enteroendocrine cells via STAT3 in human iPSC-derived Intestinal Epithelium. Zenodo. doi:10.5281/zenodo.22052476. PMC13559189 42687118

[28] Watanabe H, Du W, Son J, et al Cyb5r3-based mechanism and reversal of secondary failure to sulfonylurea in diabetes. Sci Transl Med. 2023;15(681):eabq4126. 36724243 10.1126/scitranslmed.abq4126 PMC13733829

[29] Ivanov II, Atarashi K, Manel N, et al Induction of intestinal Th17 cells by segmented filamentous bacteria. Cell. 2009;139(3):485‐498. 19836068 10.1016/j.cell.2009.09.033 PMC2796826

[30] Franceschi C, Garagnani P, Parini P, Giuliani C, Santoro A. Inflammaging: a new immune-metabolic viewpoint for age-related diseases. Nat Rev Endocrinol. 2018;14(10):576‐590. 30046148 10.1038/s41574-018-0059-4

[31] Li HJ, Ray SK, Pan N, Haigh J, Fritzsch B, Leiter AB. Intestinal Neurod1 expression impairs paneth cell differentiation and promotes enteroendocrine lineage specification. Sci Rep. 2019;9(1):19489. 31862906 10.1038/s41598-019-55292-7 PMC6925293

[32] Garbers C, Aparicio-Siegmund S, Rose-John S. The IL-6/gp130/STAT3 signaling axis: recent advances towards specific inhibition. Curr Opin Immunol. 2015;34:75‐82. 25749511 10.1016/j.coi.2015.02.008

[33] Donnelly RP, Dickensheets H, Finbloom DS. The interleukin-10 signal transduction pathway and regulation of gene expression in mononuclear phagocytes. J Interferon Cytokine Res. 1999;19(6):563‐573. 10433356 10.1089/107999099313695

[34] Sato T, Stange DE, Ferrante M, et al Long-term expansion of epithelial organoids from human colon, adenoma, adenocarcinoma, and Barrett's epithelium. Gastroenterology. 2011;141(5):1762‐1772. 21889923 10.1053/j.gastro.2011.07.050

[35] Spence JR, Mayhew CN, Rankin SA, et al Directed differentiation of human pluripotent stem cells into intestinal tissue in vitro. Nature. 2011;470(7332):105‐109. 21151107 10.1038/nature09691 PMC3033971

[36] Sinagoga KL, Wells JM. Generating human intestinal tissues from pluripotent stem cells to study development and disease. EMBO J. 2015;34(9):1149‐1163. 25792515 10.15252/embj.201490686 PMC4426477

[37] Sinagoga KL, McCauley HA, Munera JO, et al Deriving functional human enteroendocrine cells from pluripotent stem cells. Development. 2018;145(19):dev165795. 30143540 10.1242/dev.165795 PMC6198470

[38] Zeve D, Stas E, de Sousa Casal J, et al Robust differentiation of human enteroendocrine cells from intestinal stem cells. Nat Commun. 2022;13(1):261. 35017529 10.1038/s41467-021-27901-5 PMC8752608

[39] Aden K, Breuer A, Rehman A, et al Classic IL-6R signalling is dispensable for intestinal epithelial proliferation and repair. Oncogenesis. 2016;5(11):e270. 27869785 10.1038/oncsis.2016.71 PMC5141292

[40] Keir M, Yi Y, Lu T, Ghilardi N. The role of IL-22 in intestinal health and disease. J Exp Med. 2020;217(3):e20192195. 32997932 10.1084/jem.20192195 PMC7062536

[41] Bjerknes M, Cheng H. Neurogenin 3 and the enteroendocrine cell lineage in the adult mouse small intestinal epithelium. Dev Biol. 2006;300(2):722‐735. 17007831 10.1016/j.ydbio.2006.07.040

[42] Li HJ, Ray SK, Singh NK, Johnston B, Leiter AB. Basic helix-loop-helix transcription factors and enteroendocrine cell differentiation. Diabetes Obes Metab. 2011;13 Suppl 1(0 1):5‐12. 21824251 10.1111/j.1463-1326.2011.01438.x PMC3467197

[43] Gribble FM, Reimann F. Enteroendocrine cells: chemosensors in the intestinal epithelium. Annu Rev Physiol. 2016;78(1):277‐299. 26442437 10.1146/annurev-physiol-021115-105439

[44] Egerod KL, Engelstoft MS, Grunddal KV, et al A major lineage of enteroendocrine cells coexpress CCK, secretin, GIP, GLP-1, PYY, and neurotensin but not somatostatin. Endocrinology. 2012;153(12):5782‐5795. 23064014 10.1210/en.2012-1595 PMC7958714

[45] Habib AM, Richards P, Cairns LS, et al Overlap of endocrine hormone expression in the mouse intestine revealed by transcriptional profiling and flow cytometry. Endocrinology. 2012;153(7):3054‐3065. 22685263 10.1210/en.2011-2170 PMC3440453

[46] Baggio LL, Drucker DJ. Biology of incretins: GLP-1 and GIP. Gastroenterology. 2007;132(6):2131‐2157. 17498508 10.1053/j.gastro.2007.03.054

[47] Kitamoto T, Lee YK, Sultana N, et al Chemical induction of gut β-like-cells by combined FoxO1/Notch inhibition as a glucose-lowering treatment for diabetes. Mol Metab. 2022;66:101624. 36341906 10.1016/j.molmet.2022.101624 PMC9664469

[48] Du W, Wang J, Kuo T, et al Pharmacological conversion of gut epithelial cells into insulin-producing cells lowers glycemia in diabetic animals. J Clin Invest. 2022;132(24):e162720. 36282594 10.1172/JCI162720 PMC9754100

[49] Yang W, Yu T, Huang X, et al Intestinal microbiota-derived short-chain fatty acids regulation of immune cell IL-22 production and gut immunity. Nat Commun. 2020;11(1):4457. 32901017 10.1038/s41467-020-18262-6 PMC7478978

